# Poster Session II – Poster of Distinction II - A211 DIETARY FATTY ACIDS ARE ASSOCIATED WITH ANTI-GRANULOCYTE-MACROPHAGE COLONY-STIMULATING FACTOR (GM-CSF) AUTOANTIBODIES IN FIRST-DEGREE RELATIVES OF CROHN’S DISEASE PATIENTS

**DOI:** 10.1093/jcag/gwaf042.211

**Published:** 2026-02-13

**Authors:** R Khorasaniha, B Bharali, A Waslyk, S Lee, S Gnjatic, A Griffiths, P Moayyedi, H Steinhart, L Dieleman, K Jacobson, C Bernstein, H Armstrong, J Korzenik, K Croitoru, W Turpin

**Affiliations:** Lunenfeld-Tanenbaum Research Institute, Sinai Health, Toronto, ON, Canada; Lunenfeld Tanenbaum Research Institution, Sinai Health, Toronto, ON, Canada; Lunenfeld-Tanenbaum Research Institute, Sinai Health, Toronto, ON, Canada; Lunenfeld-Tanenbaum Research Institute, Sinai Health, Toronto, ON, Canada; Mount Sinai Health System, New York, NY; The Hospital for Sick Children, Toronto, ON, Canada; McMaster University, Hamilton, ON, Canada; Lunenfeld-Tanenbaum Research Institute, Sinai Health, Toronto, ON, Canada; University of Alberta, Edmonton, AB, Canada; BC Children’s Hospital, Vancouver, BC, Canada; University of Manitoba, Winnipeg, MB, Canada; University of Alberta, Edmonton, AB, Canada; Brigham and Women’s Hospital, Boston, MA; Lunenfeld-Tanenbaum Research Institute, Sinai Health, Toronto, ON, Canada; Lunenfeld-Tanenbaum Research Institute, Sinai Health, Toronto, ON, Canada

## Abstract

**Background:**

Increased neutralizing autoantibodies against granulocyte-macrophage colony-stimulating factor (GM-CSF) appear several years before Crohn’s disease (CD) diagnosis and predict complicated disease, yet the mechanisms driving these autoantibodies remain unclear. Diet shapes immune responses and gut microbiota, with macronutrients exerting immunomodulatory effects. However, the link between dietary intake and GM-CSF autoantibodies has not been investigated in healthy individuals at risk for CD.

**Aims:**

To investigate associations between dietary macronutrients (fatty acids, amino acids, and fibers) and anti-GM-CSF IgG, IgA, and IgG2 in first-degree relatives (FDRs) of CD patients.

**Methods:**

Dietary intake was assessed using a validated food frequency questionnaire (FFQ). Energy-adjusted nutrients (62 fatty acids, 19 amino acids, and 5 fiber subtypes) were computed using a previously validated diet assessment tool, the Canadian Nutrient File (CNF) and the Willett residual method. Serum GM-CSF autoantibody titers were measured by ELISA. Associations were evaluated using independent *t*-tests (high [≥100] vs. low [<100] titers) in R version 4.5.1.

**Results:**

Among 2718 individuals (52.9% female; mean age 18.5 ± 7.8 years), >100 titers were detected in 6.4% IgG, 2% IgA, and 1.2 % IgG2. No significant association was observed between titres > 100 and either fiber or amino acids. For anti-GM-CSF IgA, significant associations were observed with decreased plant sterols including campesterol (*t* = –3.39, *p* = 0.001), stigmasterol (*t* = –2.17, *p* = 0.034), total plant sterols (*t* = –2.12, *p* = 0.038), lauric acid (*t*= –2.52, *p*= 0.014) and trans-monoenoic fatty acids (*t* = 2.03, *p* = 0.047). For anti-GM-CSF IgG, significant associations were identified with decreased campesterol (*t* = –5.99, *p* = 3.8 × 10^-9^), β-sitosterol (*t* = –4.05, *p* = 6.8 × 10^-5^), stigmasterol (*t* = –3.51, *p* = 0.0005), arachidic acid (*t* = –2.68, *p* = 0.008), linoleic acid (*t* = –2.52, *p* = 0.013), total PUFA (*t* = –2.21, *p* = 0.028), total ω-6 (*t* = –2.27, *p* = 0.024), total plant sterols (*t* = –2.19, *p* = 0.029), and lignoceric acid (*t* = –2.11, *p* = 0.036). For anti-GM-CSF IgG2, significant negative associations were found for campesterol (*t* = –4.65, *p* = 2.9 × 10^-5^), β-sitosterol (*t* = –3.34, *p* = 0.002), and stigmasterol (*t* = –2.85, *p* = 0.007).

**Conclusions:**

Higher intake of plant sterols and certain fatty acids was associated with lower GM-CSF autoantibody levels, while trans fats consumption was positively associated with CD development. These findings suggest that specific dietary fats, particularly those derived from plant sources, may differently influence GM-CSF immune pathways relevant to CD risk.

**Funding Agencies:**

CCC, CIHR

